# Characterization of a *Drosophila* model to study functions of guarana seeds

**DOI:** 10.1371/journal.pone.0328985

**Published:** 2025-07-31

**Authors:** Maria Fernanda Manica-Cattani, Ivana Beatrice Mânica da Cruz, Yukiko Sato-Miyata, Lucas Siqueira Trindade, Felipe Rogalski, Euler Esteves Ribeiro, Manabu Tsuda, Toshiro Aigaki

**Affiliations:** 1 Postgraduate Program in Pharmacology, Department of Physiology and Pharmacology, Federal University of Santa Maria, Santa Maria, Rio Grande do Sul, Brazil; 2 Department of Biological Sciences, Tokyo Metropolitan University, Hachioji, Tokyo, Japan; 3 Open University of the Third Age Foundation, State University of Amazonas, Manaus, Brazil; 4 Department of Liberal Arts and Human Development, Kanagawa University of Human Services, Yokosuka-shi, Kanagawa, Japan; China Medical University, TAIWAN

## Abstract

The seeds of the Amazonian fruit, guarana (*Paullinia cupana*), have been used as traditional medicine and, in recent years, as an ingredient in commercial energy beverages. However, mechanisms underlying the beneficial effects of guarana are not well understood. To establish a model system to study molecular mechanisms underlying the beneficial effects of guarana, we investigated how its ingestion affects physiology in the fruit fly, *Drosophila melanogaster.* We found that guarana enhanced oxidative stress resistance, longevity, physical activity, and fecundity of flies. To deepen our understanding of guarana function, we performed transcriptomic, metabolomic, and fecal microbiome analyses. Transcriptomic analysis identified 58 upregulated and eight downregulated genes in guarana-fed flies. Highly upregulated genes included those encoding detoxification enzymes, such as cytochromes P450 (CYPs), glutathione S-transferases (GSTs), and Juvenile hormone epoxide hydrolase 1 (Jheh1). Metabolomic analysis identified glutathione metabolism, an antioxidant system, as being promoted by guarana ingestion. These findings likely represent the molecular basis for enhanced oxidative stress resistance and longevity in guarana-fed flies. We also analyzed fecal microbiota composition and found significant changes: guarana increased the proportion of probiotic *Lactobacillus* species, some species known to extend longevity. At the same time, it decreased the proportion of *Enterococcus faecalis*, a species known to reduce longevity. These changes might have contributed to the beneficial effects of dietary guarana. Thus, we demonstrate that guarana exerts beneficial effects in flies and provide fundamental data for further investigation of its biological mechanisms in *Drosophila*.

## Introduction

Guarana (*Paullinia cupana*), a fruit native to the Amazon basin, has been used by native Brazilians to treat high blood pressure, diarrhea, migraines, and fever [1]. In recent years, it has become a critical ingredient in commercial energy beverages [2,3]. Guarana seeds contain caffeine, an effective stimulant in higher concentrations than found in coffee beans [1]. In addition to caffeine, other xanthine alkaloids, such as theobromine and theophylline in guarana, are thought to increase its stimulant effects [4]. It also contains other bioactive compounds, including tannins, flavonoids, and saponins [1,5]. There may be complex interactions among these bioactive compounds, and other unknown substances might also contribute to the biological activity of guarana.

Several studies have demonstrated the biological activity of guarana. Guarana ingestion has been associated with an increase in basal energy expenditure [6] and weight loss [7]. Guarana has also exhibited protective effects against cancer‑related fatigue in breast cancer patients [8,9]. Guarana increased the antiproliferative effects of chemotherapeutic drugs in MCF-7 breast cancer cells [10]. It has been shown that guarana can protect cells against cadmium-induced testicular damage [11], improve memory [12], and have anti-depressant activity [13]. A case-controlled study of an elderly Amazonian population suggested that habitual guarana ingestion can reduce metabolic disorders, including hypertension, obesity, and metabolic syndrome [14]. Lower levels of cholesterol (total and low-density lipoprotein) and advanced oxidative protein product (AOPP) were found in subjects who habitually ingested guarana compared with control subjects [15]. However, the mechanistic insight into its biological activity is limited.

The fruit fly, *Drosophila melanogaster*, has been used as a model system to understand the molecular basis of human diseases [16]. It is one of the ideal model organisms in molecular nutrition research to understand the biological systems involving food-organism interactions [17]. In this study, as the first step to establishing a *Drosophila* model to study guarana function, we examined whether the biological effects of guarana could be detectable in *Drosophila*. We show that guarana intake increases oxidative stress resistance, locomotor activity, female fecundity, and female longevity in *Drosophila*. Guarana extracts significantly impact the transcriptome, metabolome, and fecal microbiome. Highly upregulated genes include those encoding detoxification enzymes, such as cytochrome P450s (CYPs), Juvenile hormone epoxide hydrolase 1 (Jheh1), and glutathione S-transferases (GSTs). Metabolomic analysis revealed that glutathione metabolism was upregulated following guarana ingestion. Thus, the enhanced anti-oxidative and detoxification systems are likely to contribute to the physiological changes induced by guarana. Notably, guarana affected the fecal microbiome, increasing the proportion of *Lactobacillus* species, some of which are known to be beneficial in *Drosophila* and other animals, including humans.

## Materials and methods

### Fly strains and culture conditions

Flies were reared at 25°C on a standard glucose-yeast medium (10% glucose, 2% yeast, 1% agar, and propionic acid to prevent fungal growth). The Canton-S strain, obtained from the Bloomington Stock Center at Indiana University was used for all experiments.

### Guarana seed powder

Toasted guarana seed powder, *Paullinia cupana* Kunth var. *sorbilis* (Mart.), was supplied by EMBRAPA Oriental (Agropecuary Research Brazilian Enterprise, Maués, Amazonas, Brazil). The chemical composition of guarana has been described previously. The concentrations of caffeine, theobromine, total catechins, and condensed tannins were 3.754, 2.065, 1.330, and 6.747 mg/g, respectively [5].

### Measurement of guarana toxicity to flies

Flies aged 5–7 days were maintained on glucose-yeast medium supplemented with guarana at concentrations of 0, 1, 5, 10, 20, 50, and 100 mg/ml for four days, and the number of surviving flies was counted. At least 120 flies were used for each condition.

### Measurement of food intake

Fifteen flies aged 5–7 days were starved for six hours, then transferred to glucose-yeast medium containing Allura Red AC dye (0.5 mg/ml) with or without 10 mg/ml of guarana powder and maintained for one hour. The flies were homogenized in 1 ml of PBS, and debris was removed by centrifugation. The amount of Allura Red AC dye in the supernatant was quantified spectrophotometrically by measuring absorbance at 503 nm.

### Paraquat resistance

The paraquat resistance assay was performed as previously described, with minor modifications [18]. Briefly, flies aged 5–7 days were fed glucose-yeast medium containing guarana powder at concentrations of 0, 5, or 10 mg/ml for 24 hours. Flies were starved for six hours and then transferred to culture vials containing medium with 15 mM paraquat. The number of dead flies was counted every 12 hours until all flies had died. Experiments were performed in triplicate, and at least 120 flies were used for each condition.

### Longevity

The lifespan of adult flies was determined as described previously [18]. Newly eclosed flies were kept on glucose-yeast medium supplemented with guarana powder at concentrations of 0, 5, or 10 mg/ml. Five flies of the same sex were placed into each chamber. The flies were transferred to fresh food, and the number of dead flies was counted every two or three days. At least 120 flies were used for each condition.

### Behavioral activity

The locomotor activity of flies was measured using the *Drosophila* Activity Monitor System (DAMS; TriKinetics, Waltham, MA, USA). Male flies aged 5 days were transferred individually to test tubes containing glucose-yeast medium supplemented with guarana powder at concentrations of 0, 5, or 10 mg/ml. Experiments were carried out at 25ºC in a humidified incubator with a 12:12 h light/dark cycle, and data were collected using TriKinetics software. At least 32 flies were used for each condition.

### Fecundity

Fecundity was assessed by counting the number of eggs laid per female. Three virgin females and three males, all aged 5 days, were placed in a vial containing standard glucose-yeast medium for 24 h. The female flies were transferred to vials containing the glucose-yeast medium supplemented with guarana powder at concentrations of 0, 5, or 10 mg/ml. The flies were then transferred to fresh vials daily, and the number of eggs laid was counted each day. At least 15 females were used to determine the fecundity for each condition.

### RNA-seq

Flies aged 5 days were used for RNA-seq analysis. After starvation for six hours, flies were transferred to glucose-yeast medium supplemented with guarana powder at concentrations of 0, 5, or 10 mg/ml for 72 h. Triplicate samples were prepared for each concentration. Total RNA was isolated from these flies using RNeasy Mini Kit (QIAGEN). RNA-seq libraries were generated using MGIEasy RNA Directional Library Prep Set (MGITech Co., Ltd.). Sequencing was performed on the DNBSEQ-G400 platform with paired-end 100 bp reads. Reads were mapped to the reference sequence (*Drosophila melanogaster version 6 reference genome;* *https://www.ncbi.nlm.nih.gov/assembly/GCF_000001215.4/)* using the HISAT2 (version 2.2.1) and transcript abundance was quantified as TPM (transcripts per million mapped reads) using featureCounts (version 2.0.0). Differential expression analysis was carried out using edgeR (version 3.28.1). Genes displaying a significant *p*-value<0.05 and at least a 2-fold change were used for further analysis. The sequence data are available in the DDBJ under accession numbers DRR355795-DRR355803.

### Quantitative real-time PCR

Five-day-old flies were starved for six hours and then transferred to glucose-yeast media supplemented with 0, 5, or 10 mg/ml guarana for 72 hours prior to RNA-seq analysis. Total RNA was isolated using TRIzol Reagent (Invitrogen), treated with DNase, and reverse-transcribed using the SuperScript IV VILO Master Mix (Invitrogen). Quantitative PCR was performed using SYBR Premix Ex Taq (Takara, Shiga, Japan) on a Chromo4 Detector (Bio-Rad) with the following primers for *rp49*: forward 5’-GCTAAGCTGTCGCACAAATG-3’ and reverse 5’-TGTGCACCAGGAACTTCTTG-3’, *Cyp12d1*: forward 5’- CGGAATACACCAGTGCCATG −3’ and reverse 5’- AATCCTTGCGGCCAAACATT-3’, *Cyp6w1*: forward 5’- CCACAAAGTTCATCCGGTCC-3’ and reverse 5’- GTCCTTTCGCGCTCCTTTAG-3’, *Cyp6g1*: forward 5’-GGACAGCATAGCCACGATTG-3’ and reverse 5’-ACTCTGCGTTGGGATTCTCT-3’, *MtnC:* forward 5’-GGCCCCAAAGATCAGTGTTG-3’ and reverse 5’-GGCTTACACAGGGGCTATCA-3’, *GSTD5*: forward 5’-ATGACACCCTCTTCCCCAAG-3’ and reverse 5’-TCGAAGTAGAGGCGTTGGTT-3’, and *Jheh1*: forward 5’- CATTGCCACCTTGTATCCGG-3’ and reverse 5’- TAAACCCTTAGCCAGCGACT-3’. Expression levels were normalized to *rp49* transcript levels in each sample. Triplicate biological samples were prepared for each guarana concentration.

### Metabolomic analysis

Flies aged 5 days were used for metabolomic analysis. After starvation for six hours, flies were transferred to glucose-yeast medium supplemented with guarana powder at concentrations of 0, 5, or 10 mg/ml for 72 h. Flies were weighed and homogenized in 75% acetonitrile (20 µl of solvent per fly). The homogenates were centrifuged to obtain the supernatant. Metabolites were measured using a Waters UPLC-Xevo QTof MS system (Waters, Milford, MA, USA) as previously described [19]. In brief, samples were separated on an Acquity UPLC HSS T3 column (2.1 Å, 100 µm, 1.8 µm) and detected using a dual electrospray ionization probe operated in both negative and positive ion modes. The raw data were processed using MarkerLynx and QuanLynx software (Waters) for feature alignment, signal detection, and signal integration. Annotated metabolites were identified based on their retention time, m/z ratio, and comparison with the MS/MS spectra of 117 reference standards. All statistical analyses of metabolomic data were performed using MetaboAnalyst 3.0 (Xia et al. 2015), with data auto-scaled to unit variance using R version 3.3.1 (The R Project for Statistical Computing: https://www.R-project.org/). Selected metabolites were analyzed by the unpaired Student’s *t*-test.

### Metagenomic analysis of fecal microbiome

Flies aged 5 days were maintained in glucose-yeast medium supplemented with guarana powder at concentrations of 0, 5, or 10 mg/ml for 72 hours. Flies were transferred to a clean medium vial for 24 hours. The fecal spots adhering on the vial walls were collected with sterile buffer-saturated swabs. Genomic bacterial DNA was isolated from the feces using the PowerSoil DNA isolation Kit (MoBio Laboratories, Carlsbad, CA). The V3–V4 regions of the 16S rRNA genes were amplified using the two-step tailed-PCR method. (1st-27F mod MIX; 5’-ACACTCTTTCCCTACACGACGCTCTTCCGATCT-NNNNN-AGRGTTTGATYMTGGCTCAG-3’, 1st-338R_MIX; 5’-GTGACTGGAGTTCAGACGTGTGCTCTTCCGATCT-NNNNN-TGCTGCCTCCCGTAGGAGT3’, 2nd-F; 5’-AATGATACGGCGACCACCGAGATCTACAC-Index2-ACACTCTTTCCCTACACGACGC-3’, 2nd-R; 5’-CAAGCAGAAGACGGCATACGAGAT-Index1-GTGACTGGAGTTCAGACGTGTG-3’) The PCR products were quantified using a Synergy H1 reader and the QuantiFluor dsDNA System, and the quality of the libraries was confirmed using a Fragment Analyzer System with a dsDNA 915 Reagent Kit (Advanced Analytical Technologies, Inc., Ankeny, IA, USA). Sequencing was performed using the Illumina MiSeq platform with paired-end 300 bp reads. Obtained sequences were processed using the QIIME 2 (version 2021.4) and clustered into operational taxonomic units (OTUs) based on similarity. The OTUs were annotated using the NCBI BLAST and subjected to diversity and taxonomy analyses. The sequence data are available in the DDBJ under accession numbers DRR355804-DRR355812.

## Results and discussion

### Effects of guarana on the viability of flies

**Fig 1:**
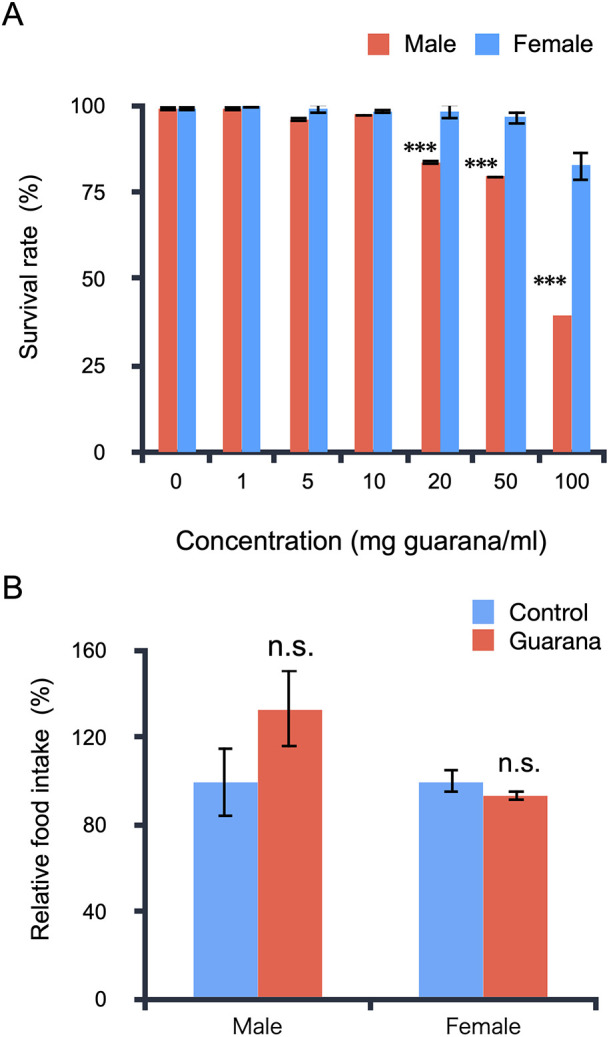
Effects of guarana on fly viability and food intake. (A) Effects of guarana on the viability of adult flies. Adult flies were maintained on medium supplemented with the indicated concentrations of guarana. There were no significant effects on viability up to 10 mg/ml in both sexes. Results were compared with controls (0 mg guarana/ml) for each sex group. Data represent the mean ± SE of at least three experiments (*post hoc* Dunnett’s test, *: p < 0.05, ***: p < 0.001). (B) Effects of guarana on food intake. Food intake was measured spectrophotometrically using flies fed medium containing 0 (control) or 10 mg/ml guarana. Allura Red AC dye was added to medium to monitor food intake. Data represent the mean ± SE of at least three experiments (*t*-tes*t*, n.s., not significant).

We first examined whether guarana intake could affect the viability of flies. Flies were maintained on medium containing various concentrations of guarana for 96 hours. Afterward, we counted the number of dead flies. There was no significant reduction in the survival rate of flies at concentrations up to 10 mg/ml for males and 50 mg/ml for females. However, the survival rate was significantly decreased at 20 mg/ml for males and 100 mg/ml for females (Fig 1A). The reduction in survival was dose-dependent in both sexes, but male flies were more sensitive to the toxicity of guarana than females. These results indicate that guarana exhibits dose-dependent toxicity in flies at high concentrations.

Guarana seeds have been shown to contain bitter-tasting compounds, such as theophylline, tannins, and theobromine [5]. As fruit flies exhibit repulsive responses to bitter foods, we examined whether guarana affects food intake. After starvation for six hours, flies were transferred to medium containing red food dye with or without guarana powder (10 mg/ml) and allowed to feed for one hour. Then, the flies were homogenized, and the amount of ingested dye was quantified via spectrophotometry. There was no significant difference in the amount of ingested dye between flies fed medium with and without guarana for both sexes, indicating that guarana supplementation did not affect the amount of food ingested (Fig 1B).

### Guarana increased the paraquat resistance and longevity of flies

**Fig 2:**
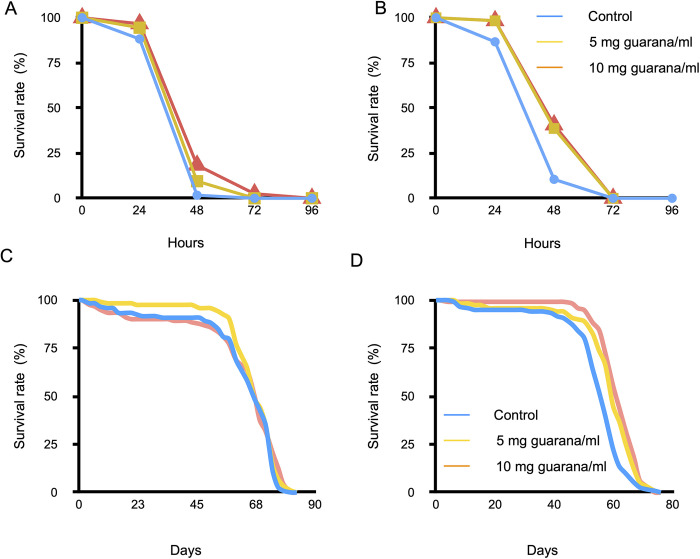
Effects of guarana on paraquat resistance and longevity. Survival curves of male (A) and female (B) flies exposed to 15 mM paraquat following pretreatment with 0 (control), 5, or 10 mg/ml guarana. The mean survival periods of the guarana-fed flies were significantly extended compared to control flies (Log-rank test, p < 0.001). Longevities of male (C) and female (D) flies were measured with glucose-yeast medium containing 0 (control), 5, or 10 mg guarana/ml. Continuous guarana intake significantly extended the longevity of female flies (Log-rank test, p < 0.001) but had no effect on males.

It has been demonstrated that guarana extracts have antioxidant and radical-scavenging activity *in vitro* [20]*.* To examine whether dietary guarana could affect oxidative stress resistance in flies, we measured the survival rate of guarana-fed flies exposed to paraquat as an intracellular generator of reactive oxygen species. We selected the concentrations of guarana based on the toxicity assessments to avoid confounding effects resulting from its toxicity. Specifically, 5 mg/ml was chosen as the lowest concentration that showed no significant adverse effects, and 10 mg/ml as the highest non-toxic dose that balanced efficacy and safety. These concentrations allowed us to evaluate the effects of guarana on oxidative stress resistance without the interference from its toxicity-related mortality. Flies were maintained in standard glucose-yeast medium supplemented with guarana powder (0, 5, or 10 mg/ml) for 24 hours and then transferred to a medium containing 15 mM paraquat. The mean (± SE) survival periods of flies fed with 5 and 10 mg/ml guarana (males: 32.9 ± 12.4 and 33.4 ± 12.4 h, females: 24.9 ± 7.3 and 28.0 ± 12.1 h, respectively) were significantly more extended than that of control males (22.7 ± 12.1 h) and females (21.6 ± 8.5 h), for both sexes and both concentrations (p < 0.001) (Fig 2A and B). These findings suggest that guarana supplementation may contribute to increased resistance against paraquat-induced oxidative stress.

Resistance to oxidative stress is often correlated with the longevity of adult flies [21]. Thus, we next determined whether dietary guarana could affect longevity of flies. We measured the longevity using glucose-yeast medium with or without guarana supplementation. There was no significant increase in the mean longevity of males for the guarana-fed flies. The mean (± SE) longevity of male flies fed 0 (control), 5, and 10 mg/ml of guarana was 71.7 ± 2.4, 71.2 ± 4.4, and 74.1 ± 3.4 days, respectively (Fig 2C). In contrast, female flies fed guarana exhibited a significant increase in mean longevity compared with control flies (p < 0.05). The mean (± SE) longevity of female flies was 61.3 ± 4.9, 64.6 ± 4.2, and 65.2 ± 4.0 days for 0 (control), 5, and 10 mg/ml of guarana, respectively (Fig 2D). The absence of a significant effect in males might be due to a higher sensitivity to guarana’s toxicity, which could potentially mask any lifespan-extending effects in this sex. Additional factors may also contribute to the observed sex-specific differences, as discussed later.

### Guarana increased the locomotor activity and the fecundity of flies

**Fig 3:**
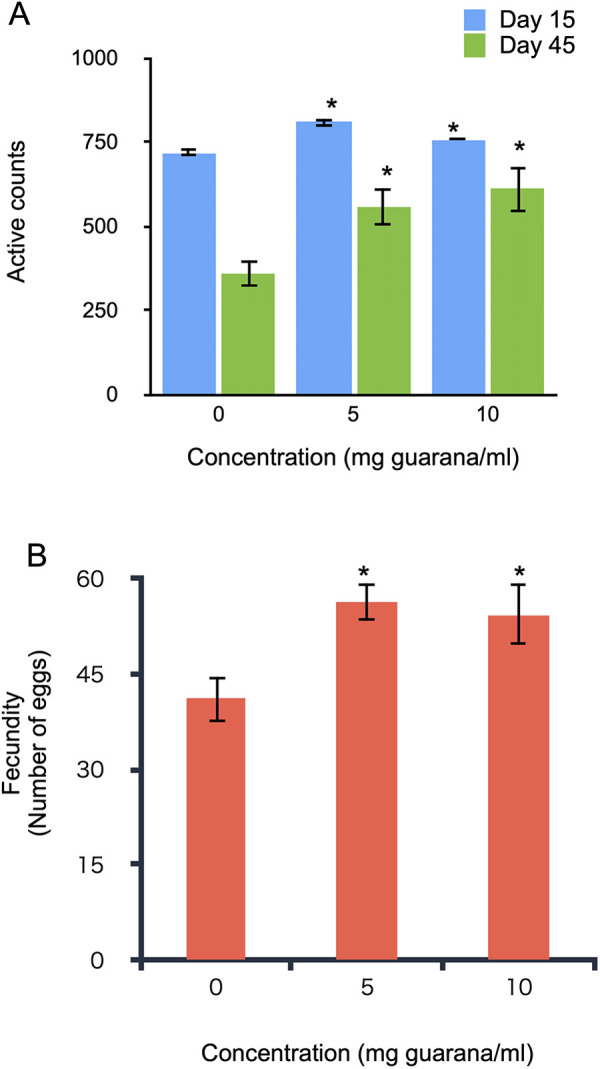
Effects of dietary guarana on physical activity and fecundity in flies. (A) Effects of guarana intake on the physical activity of flies. Physical activity was measured in flies maintained on glucose-yeast medium supplemented with varying concentrations of guarana. Activity levels were compared with controls (0 mg/ml guarana) within each age group. (B) Effects of guarana intake on fecundity (number of laid eggs). Flies fed guarana laid more eggs than control flies. Data represent the mean ± SE (t-test, *: p< 0.05, **: p<0.01).

We examined the impact of habitual guarana intake on the locomotor activity of flies. Flies were maintained on glucose-yeast medium supplemented with 0, 5, or 10 mg/ml guarana for 15 or 45 days, and then locomotor activity was measured individually using the DAMS. In this system, the activity of a single fly placed in a glass tube was estimated by counting the number of times the fly breaks an infrared beam across the tube. Fifteen-day-old flies fed guarana were significantly more active than control flies (Fig 3A). The mean active counts of young flies fed with 5 and 10 mg/ml of guarana increased by 12.3% and 5.5%, respectively, compared with control flies. The difference in the activity counts between the guarana-supplemented and control flies was more remarkable in 45-day-old flies than in 15-day-old flies (Fig 3A). The mean active counts of the older flies fed with 5 and 10 mg/ml of guarana increased by 53.8% and 68.9% compared with the control flies. These results indicate that continuous intake of guarana significantly increased the physical activity of flies.

We next investigated the effect of guarana on female fecundity. We kept mated females in a vial containing glucose-yeast medium supplemented with 0, 5, and 10 mg/ml of guarana powder. Flies were transferred to fresh medium daily, and the number of eggs was counted every day until the females stopped laying eggs. The total egg production was significantly increased in the guarana-fed flies (Fig 3B). The average (± SE) number of eggs laid per female was 41.1 ± 3.7, 56.4 ± 2.9, and 54.4 ± 5.0 for 0, 5, and 10 mg/ml of guarana, respectively. These results indicate that sustained guarana intake enhances reproductive output in females.

Guarana contains high levels of caffeine and its derived metabolites, including theobromine and theophylline. Caffeine has been shown to activate two dorsal-paired medial neurons and promotes egg-laying [22]. Caffeine and related metabolites in guarana may be responsible for the promotion of the female egg-laying behavior. It is also possible that these compounds are involved in the female-specific extension of lifespan.

### Effects of guarana on the transcriptome

**Fig 4:**
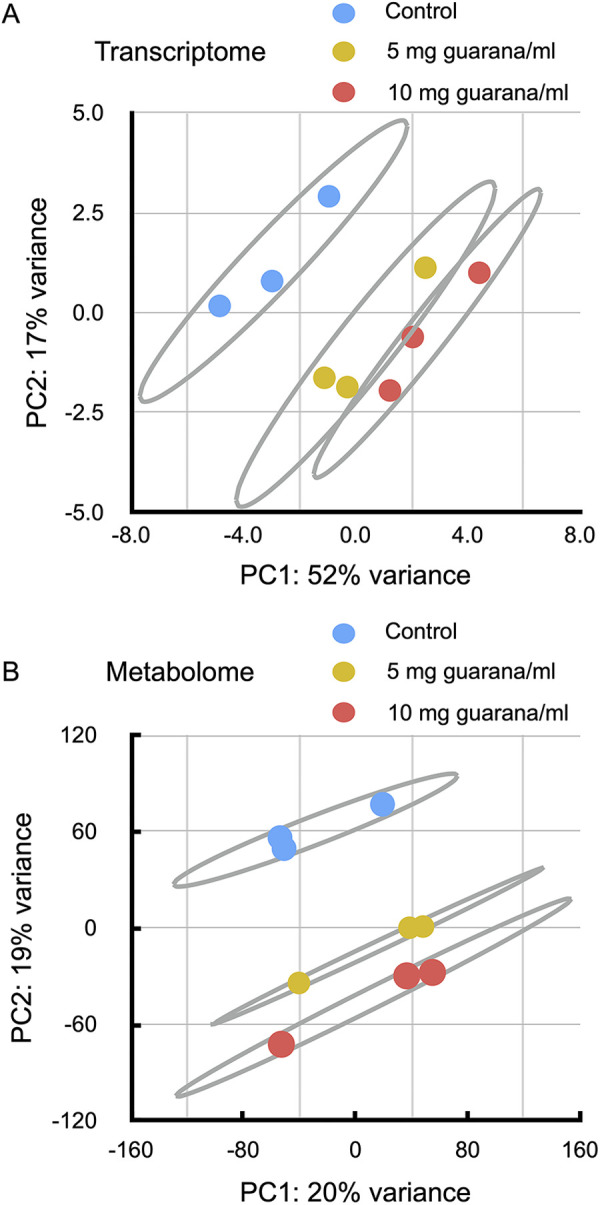
Effects of guarana intake on transcriptomic and metabolomic profiles in flies. Principal component analysis (PCA) of transcriptomic (A) and metabolomic (B) profiles with 95% confidence ellipses. Three distinct clusters were segregated according to the concentration of guarana in both analyses.

To explore molecular genetic bases of the guarana-induced phenotypic changes, we analyzed the effects of guarana intake on gene expression in flies. Flies were kept on the glucose-yeast medium supplemented with guarana (0, 5, or 10 mg/ml) for 72 hours and were subjected to RNA-seq analysis. Principal component analysis revealed three distinct clusters corresponding to the guarana concentration, suggesting that guarana intake is associated with dose-dependent changes in gene expression profiles (Fig 4A).

**Fig 5:**
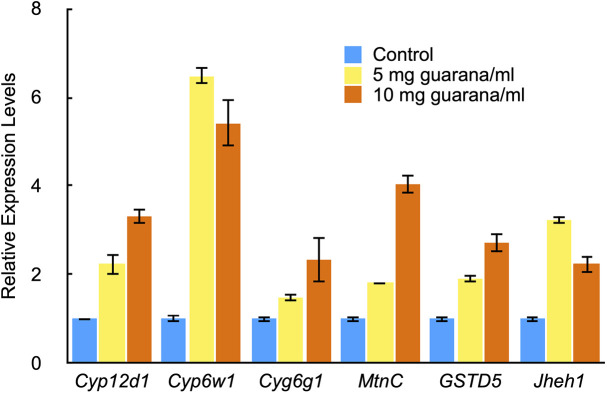
Cross-validation of increased expression levels from RNAseq data using RT-PCR. The expression levels of the indicated genes, as confirmed by RNA-seq data, were further assessed by quantitative RT-PCR after 72 hours of guarana treatment. Expression levels of the *rp49* gene were used as an internal control. The results are represented by blue (0 mg/ml guarana), yellow (5 mg/ml guarana), and orange (10 mg/ml guarana) bars. Mean (± S.E.) values were calculated from three independent experiments for each gene. The expression levels of all genes were significantly increased upon guarana treatment (*t-*test; *p* < 0.01).

A total of 58 genes were upregulated, and eight were downregulated following guarana treatment with at least a 2-fold change at either guarana concentration (S1 Table). Using real-time PCR, we confirmed that all five selected genes from this set were upregulated in response to guarana intake, thereby supporting the RNA-seq data (Fig 5). Next, gene set enrichment analysis was performed, which suggested that genes involved in detoxification processes were significantly upregulated in guarana-fed flies (Table 1). These enzymes, including cytochrome P450s (CYPs) and glutathione S-transferases (GSTs), may contribute to enhanced oxidative stress resistance and potentially to lifespan extension. As females have been reported to be relatively more responsive to antioxidant enzyme expression [23,24], this difference may partly explain the sex-specific effects observed in guarana-fed flies.

**Table 1:** List of Gene Ontology Terms significantly altered by dietary guarana.

GO biological process	Fold Enrichment	Raw *p*-Value	FDR	Genes
Response to DDT (GO:0046680)	> 100	4.58E-11	3.57E-07	*Cyp12d1-d; Cyp12d1-p; Cyp6w1; Cyp6g1; Cyp6a2*
Response to insecticide (GO:0017085)	> 100	1.48E-09	5.77E-06	*Cyp12d1-d; Cyp12d1-p; Cyp6w1; Cyp6g1; Cyp6a2*
Response to organic cyclic compound (GO:0014070)	24.2	2.14E-06	3.33E-03	*Cyp12d1-d; Cyp12d1-p; Cyp6w1; Cyp6g1; Cyp6a2*
Glutathione metabolic process (GO:0006749)	18.98	5.29E-03	1.00E + 00	*GstE3; GstD5*
Response to metal ion (GO:0010038)	17.95	5.87E-03	1.00E + 00	*MtnC; Cyp6g1*
Carbohydrate metabolic process (GO:0005975)	15.99	2.68E-08	6.95E-05	*Mal-A8; Mal-A7; Mal-A6; Mal-A1; Mal-B1; Mal-A2; tobi; Amy-d*
Chorion-containing eggshell formation (GO:0007304)	8.03	2.62E-02	1.00E + 00	*Cp7Fa; Muc12Ea*

Among the upregulated detoxification genes, we identified *Jheh1*, which encodes an epoxide hydrolase involved in the detoxification of epoxides in mammals. Although the juvenile hormone (JH) esterase-like gene family comprises numerous members, only a small proportion of these genes in *Lepidoptera* possess JH-degrading activity [25]. Phylogenetic analyses suggest that this activity evolved in *Lepidoptera* after their divergence from *Dipteran* insects including *Drosophila*. Moreover, functional studies suggest that *Drosophila* Jheh1 lacks JH-degrading activity [26]. Therefore, the upregulation of *Jheh1* is more likely associated with enhanced detoxification or oxidative stress responses rather than direct modulation of JH signaling. Nevertheless, a potential role in JH metabolism cannot be entirely excluded. Notably, JH has been implicated in lifespan regulation in flies, functioning downstream of insulin signaling, with particularly pronounced effects in females [27]. Thus, it remains possible that guarana-induced, female-specific lifespan extension involves mechanisms related to JH signaling, warranting further investigation.

*Metallothionein C* (*MtnC*) and *Cyp6g1* were upregulated in the guarana-fed flies (Table 1 and S1 Table). These genes are thought to be involved in protection against both xenobiotic heavy metals, such as mercury, cadmium, and oxidative stress [28]. Interestingly, it has been demonstrated that guarana supplementation significantly reduced cadmium-induced damage in Wister rat testis [29]. Although the molecular mechanism by which guarana reduces cadmium toxicity is still unclear, our results suggest the possibility that metallothionein enzymes may be involved in the protection against the toxicity of heavy metals.

The gene set enrichment analysis implied that the genes *Muc12Ea* and *Cp7fa* participating in chorion-containing eggshell formation pathway were upregulated in guarana-fed flies (Table 1). In addition, expression of genes encoding putative eggshell proteins, including *CG32642*, *CG13083*, and *CG13084,* was also increased in guarana-fed flies (S1 Table). The fecundity test indicated a significant increase in the number of eggs laid, suggesting that guarana stimulates both egg production and egg-laying behavior. Further functional studies are needed to clarify the causal roles of these gene expression changes.

### Effects of guarana on the metabolome

To characterize the effects of guarana on metabolism, we analyzed the metabolomic profiles of flies using liquid chromatography with tandem mass spectrometry (LC-MS/MS). Flies were kept in glucose-yeast medium supplemented with 0, 5, or 10 mg/ml guarana for 72 hours. Principal component analysis revealed clear separation among the groups, and the second principal component (PC2) scores were negatively correlated with the guarana concentration (Fig 4B), suggesting a dose-dependent effect on metabolomic profiles. We detected 9902 unique LC-MS/MS peaks and determined that 52 metabolites were significantly increased in the 10 mg/ml guarana-treated flies (>1.5-fold, p < 0.05). We performed metabolite set enrichment analysis using Metaboanalyst 3.0, which identifies enriched pathways based on the number of affected metabolites [30,31]. The analysis revealed that guarana intake affected various metabolic pathways, such as protein biosynthesis, amino acid metabolism, ammonia recycling, the urea cycle, glutathione metabolism, and the citric acid cycle (Table 2). Among the altered metabolic pathways, glutathione metabolism is likely involved in oxidative stress resistance and longevity [31]. Other pathways may be related to the physiological phenotype of guarana-fed flies, but the specific connection remains unclear.

**Table 2:** The list of metabolic pathways significantly changed by dietary guarana.

	Fold Enrichment	Raw p-Value	FDR
Protein Biosynthesis	15.6	3.42E-10	2.73E-08
Glycine, Serine, and Threonine Metabolism	6.37	0.000724	0.029
Ammonia Recycling	7.33	0.00152	0.0405
Urea Cycle	6.59	0.00231	0.0412
Glutathione Metabolism	9.9	0.00258	0.0412
Histidine Metabolism	8.98	0.00347	0.0453
Citric Acid Cycle	5.73	0.00396	0.0453

### Effects of guarana on the fecal microbiome

**Fig 6:**
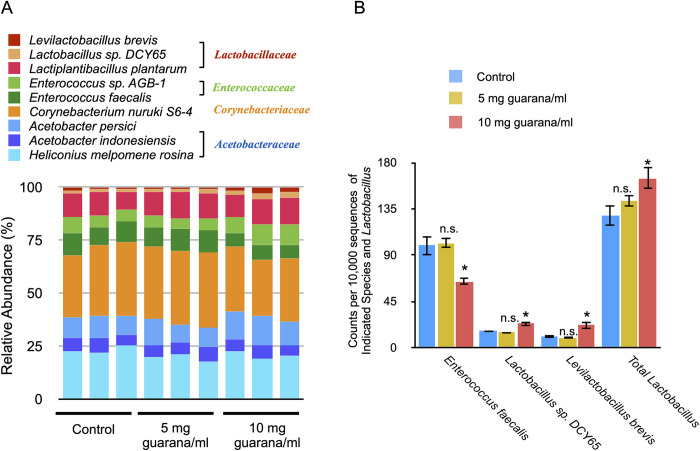
Effects of dietary guarana on the microbiota composition in flies. (A) Microbiota composition of flies fed different concentrations of guarana. Each column represents a biological replicate with three replicates analyzed per group. The stacked bar chart shows the relative abundance of identified bacterial species, with species names listed above the chart. (B) The 16S rRNA-based relative abundance of indicated species and Lactobacillus was calculated based on counts per 10,000 reads. Data represent the mean ± SE of at least three experiments (t-test, *: p < 0.05, n.s., not significant). Flies were maintained on glucose-yeast medium with indicated concentrations of guarana for 72 hours. Bacterial genomic DNA was isolated from fly feces and the V3–V4 regions of the 16S rRNA genes were amplified and sequenced, and clustered into operational taxonomic units (OTUs) based on sequence similarity.

*Drosophila* has been used as a simple animal model for microbiome research [32]. Since the microbiota has been shown to affect fly physiology, we tested whether guarana intake modulates the microbiota in flies. We performed the 16S rRNA amplicon sequencing to characterize bacterial communities in flies. Flies were fed glucose-yeast medium with guarana (0, 5, or 10 mg/ml) for 72 hours and bacterial genomic DNA was isolated from the fecal samples from those flies [33]. The V3–V4 variable regions of the bacterial 16S rRNA genes were amplified by two-step PCR and subjected to high-throughput sequencing. The obtained sequences were aligned against the NCBI BLAST to identify species and characterize the bacterial communities in flies. In general, fly microbiota exhibits relatively low species diversity compared to humans [34]. *Acetobacteraceae*, *Lactobacillales*, and *Enterococcaceae* species have been shown to dominate in laboratory strains of *Drosophila melanogaster* [34]*.* In addition to these species, *Corynebacteriales* species, typically found in the fly gut [35], were present in our laboratory flies (Fig 6A).

**Fig 7:**
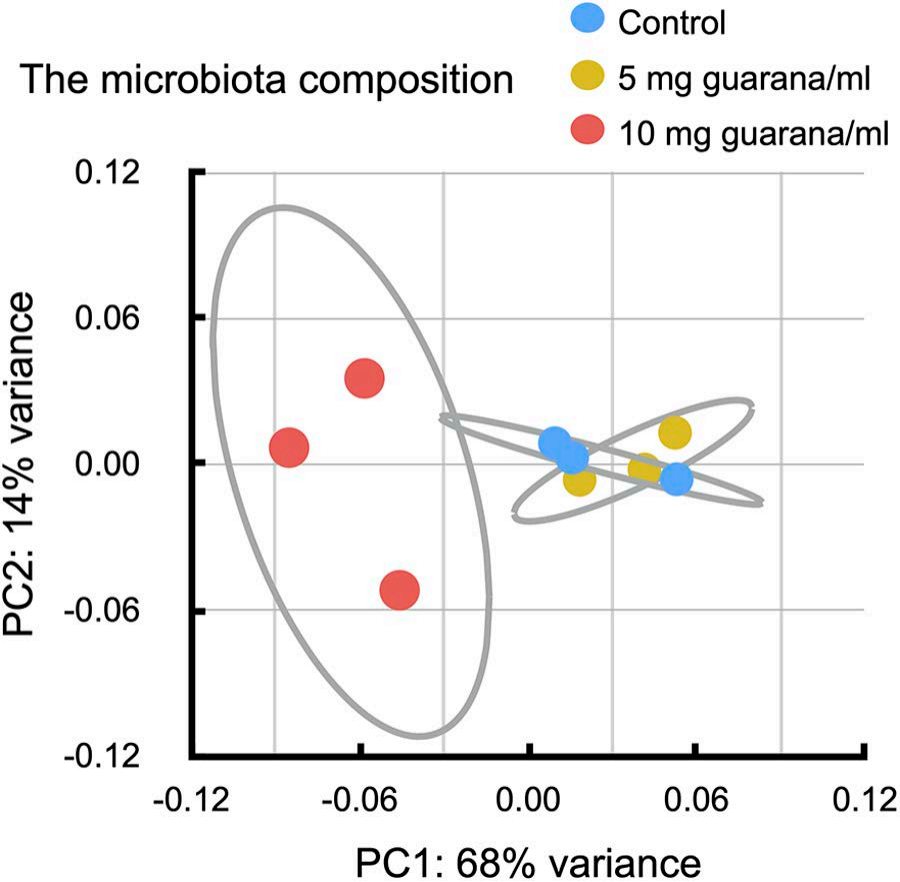
Impacts of guarana ingestion on the microbiota composition of flies. Principal coordinate analysis (PCoA) of the microbiota composition was performed using the Bray-Curtis similarity index, and 95% confidence ellipses were drawn. The microbiota composition of flies fed 10 mg/ml guarana was significantly different from that of the other two groups (control and 5 mg/ml).

To compare the microbiome profiles, we performed principal coordinate analysis (PCoA) with the Bray-Curtis distances (Fig 7). There was no apparent difference in the microbiota composition between control flies and those maintained in a medium supplemented with guarana at 5 mg/ml. In contrast, flies fed 10 mg/ml guarana exhibited a distinct clustering pattern, clearly separated from the control and 5 mg/mL groups. Alpha diversity metrics, including the Shannon and Chao1 indices, showed no significant differences between control and flies fed 10 mg/ml guarana.

To obtain a comprehensive and robust overview, we performed LEfSe analysis at the genus level. However, this approach did not identify significant differences between control and guarana-fed flies, likely due to the conservative detection threshold of the method. To explore more specific taxonomic shifts, we conducted pairwise comparisons at the species level. Despite the limited statistical power associated with the small sample size (n = 3), unpaired t-tests revealed significant increases in the relative abundance of *Levilactobacillus brevis* (formerly *Lactobacillus brevis*), *Lactobacillus* sp. DCY65, and overall *Lactobacillus* abundance in flies fed 10 mg/ml guarana (Fig 6B). *Lactobacilli* are widely regarded as probiotics, promoting health and longevity in humans. Notably, supplementation of *Lactobacillus reuteri* extended longevity in flies [36]. Similarly, there was a significant increase in longevity in flies treated with *Lactobacillus fermentum* [37]. Conversely, the relative abundance of *Enterococcus faecalis*, a species known to reduce lifespan in *Drosophila* [38], was significantly decreased. These findings suggest that guarana-induced shifts in the gut microbiota, particularly the enrichment of beneficial *Lactobacillus* species and the reduction of potentially harmful taxa, may contribute to the observed physiological effects. However, further functional studies are required to establish direct causal relationships.

## Conclusion

To establish a model organism for studying molecular mechanisms underlying the beneficial effects of the Amazonian fruit guarana (*Paullinia cupana*), we investigated its effects in the fruit fly, *Drosophila melanogaster*. Guarana ingestion conferred several benefits in flies, including increased oxidative stress resistance, extended lifespan, enhanced physical activity, and higher female fecundity. These effects may be partly due to rich content of radical-scavenging compounds in guarana, such as catechins, epicatechins, tannins, and proanthocyanidols [5], which likely contribute to enhanced oxidative stress resistance and increased longevity observed in guarana-fed flies. Among the components in guarana, caffeine and catechins have been shown to extend the lifespan of flies in a PGRP-LB mutant model of neurodegeneration and under paraquat-induced oxidative stress, respectively [39,40]. However, it is important to note that while these associations suggest potential benefits, the observed physiological effects remain correlated with the presence of specific compounds, rather than providing direct evidence of causation. Whether the effects result from additive or synergistic actions of these compounds, or other bioactive components, is still unclear. To address these issues, future studies using fractionated extracts or testing individual components are needed to clarify their specific contributions and establish causal relationships.

To understand the molecular events associated with guarana ingestion, we performed transcriptomic and metabolomic analyses. Guarana enhanced the expression of genes involved in detoxification pathways, such as cytochromes P450 enzymes (CYPs), glutathione S-transferases (GSTs), and Juvenile hormone epoxide hydrolase 1 (Jheh1). Metabolomic analysis identified glutathione metabolism, an antioxidant system, as one of the affected pathways. However, while these molecular changes correlate with the observed physiological effects, it remains to be determined whether they directly mediate these effects or are secondary consequences. Further functional studies, such as gene knockout experiments, would be valuable to elucidate the causal relationships among gene expression changes, metabolic alterations, and physiological effects. We also found significant changes in the composition of fecal microbiota. Guarana increased the proportion of probiotic *Lactobacillus* species, some of which are known to extend longevity. In addition, guarana decreased the proportion of *Enterococcus faecalis,* a species associates with reduce longevity. While the microbiota shifts observed were consistent with the beneficial phenotypes in guarana-fed flies, direct causality has not been established.

In summary, our findings lay the groundwork for further exploration of the physiological and molecular mechanisms underlying the beneficial effects of guarana in *Drosophila.* Giving that guarana enhances oxidative stress resistance and detoxification systems, it may offer protective effects against human diseases. These possibilities can be readily explored in *Drosophila*, where various disease models are available.

## Supporting information

S1 Table List of genes whose expression was altered by guarana ingestion. (DOCX)

S2 Table List of metabolites affected by guarana ingestion. (DOCX)
