# Supplementary material for: Characterization of a Drosophila model to study functions of guarana seeds
Source: PLoS One. 2025 Jul 31;20(7):e0328985. doi: 10.1371/journal.pone.0328985 (PMC12312877; doi:10.1371/journal.pone.0328985)
Supplement: S1 Table — (DOCX) [file pone.0328985.s001.docx]

| **Genes** | **Log2 Fold Change** | | **Description** |
| --- | --- | --- | --- |
|  | **5 mg/ml Guarana** | **10 mg/ml Guarana** |  |
| *Cyp6w1* | 3.3 | 7.4 | Cyp6w1 |
| *CG11893* | 1.4 | 3.5 | Ecdysteroid kinase-like |
| *Cyp6d5* | 2 | 3.2 | Cyp6d5 |
| *Cyp12d1-d* | 2 | 3 | Cyp12d1-d |
| *CG4377* | 1.1 | 2.8 | Uncharacterized protein |
| *CG32642* | 1.4 | 2.7 | Uncharacterized protein |
| *Mal-A7* | 1.6 | 2.5 | Maltase A7 |
| *CG43349* | 1.4 | 2.2 | Uncharacterized protein |
| *CG13084* | 1.5 | 2.2 | Uncharacterized protein |
| *Cyp9b2* | 1.8 | 2.1 | Cytochrome P450-9b2 |
| *CG6484* | 1.3 | 2.1 | Uncharacterized protein |
| *Cyp6g1* | 1.3 | 2.1 | Cyp6g1 |
| *Mal-A1* | 1.3 | 2.1 | Maltase A1 |
| *Amy-d* | 1.3 | 2 | Amylase distal |
| *Mal-A8* | 1.2 | 2 | Maltase A8 |
| *CG43055* | 1.3 | 2 | C-type lectin-like |
| *CG43348* |  | 4.4 | Uncharacterized protein |
| *CG34040* |  | 3.3 | Uncharacterized protein |
| *Cyp6a2* |  | 3.2 | Cytochrome P450-6a2 |
| *Cyp309a1* |  | 3 | Cyp309a1 |
| *CG3513* |  | 2.9 | Uncharacterized protein |
| *CG31266* |  | 2.9 | Peptidase S1, PA clan |
| *Cyp12d1-p* |  | 2.8 | Cyp12d1-p |
| *CG6508* |  | 2.8 | Aspartic peptidase A1 family |
| *Mal-B1* |  | 2.8 | Maltase B1 |
| *Ugt35C1* |  | 2.7 | UDP-glycosyltransferase family 35 member C1 |
| *CG31104* |  | 2.7 | Ecdysteroid kinase-like |
| *CG6830* |  | 2.4 | Ecdysteroid kinase-like |
| *CG31267* |  | 2.4 | Peptidase S1, PA clan |
| *MtnC* |  | 2.3 | Metallothionein C |
| *CG13083* |  | 2.3 | Uncharacterized protein |
| *Alp9* |  | 2.2 | Alkaline phosphatase 9, transcript variant B |
| *CG17475* |  | 2.2 | Peptidase S1, PA clan |
| *CG3739* |  | 2.2 | S28 Serine exopeptidase |
| *CG9360* |  | 2.2 | Short-chain dehydrogenase/reductase SDR |
| *CG33514* |  | 2.1 | CRAL/TRIO, N-terminal domain |
| *GstD5* |  | 2.1 | Glutathione S transferase D5 |
| *CG31300* |  | 2.1 | Ecdysteroid kinase-like |
| *mesh* |  | 2.1 | Mesh |
| *Muc12Ea* |  | 2 | Mucin 12Ea |
| *Alp10* |  | 2 | Alkaline phosphatase 10 |
| *tobi* |  | 2 | Target of brain insulin |
| *Mal-A2* |  | 2 | Maltase A2 |
| *CG15784* | 1.6 |  | Activity-regulated cytoskeleton associated protein 1 |
| *AOX1* | 1.4 |  | Aldehyde oxidase 1 |
| *Jheh1* | 1.4 |  | Juvenile hormone epoxide hydrolase 1 |
| *GstE3* | 1.3 |  | Glutathione S transferase E3 |
| *Arc1* | 1.3 |  | Activity-regulated cytoskeleton associated protein 1 |
| *CG31288* | 1.3 |  | Ecdysteroid kinase-like |
| *CG18179* | 1.2 |  | Peptidase S1, PA clan |
| *CG18180* | 1.2 |  | Peptidase S1, PA clan |
| *Cp7Fa* | 1.2 |  | Chorion protein a at 7F |
| *CG15570* | 1.1 |  | Uncharacterized protein |
| *CG15423* | 1.1 |  | Uncharacterized protein |
| *Mal-A6* | 1.1 |  | Maltase A6 |
| *CG9672* | 1.1 |  | Peptidase S1, PA clan |
| *CG7542* | 1 |  | Peptidase S1, PA clan |
| *CG42825* | 1 |  | MFS transporter superfamily |
| *CG13947* |  | -1.4 | Uncharacterized protein |
| *BomBc1* | -2.4 |  | Bomanin bicipital 1 |
| *Mtk* | -1.6 |  | Metchnikowin |
| *Act88F* | -1.5 |  | Actin 88F |
| *Elo68alpha* | -1.1 |  | Elongase 68alpha |
| *CG45045* | -1.1 |  | Uncharacterized protein |
| *BomS1* | -1.1 |  | Bomanin short 1 |
| *CG44142* | -1 |  | Uncharacterized protein |
