# Supplementary material for: Characterization of a Drosophila model to study functions of guarana seeds
Source: PLoS One. 2025 Jul 31;20(7):e0328985. doi: 10.1371/journal.pone.0328985 (PMC12312877; doi:10.1371/journal.pone.0328985)
Supplement: S2 Table — (DOCX) [file pone.0328985.s002.docx]

| **Metabolite** | **Log2 Fold Change** |
| --- | --- |
| Adenosine triphosphate | 1.3 |
| Trehalose | 1.3 |
| NAD | 1.3 |
| NADPH | 1.3 |
| D-Glucose | 1.3 |
| NADH | 1.3 |
| L-Tyrosine | 1.4 |
| Octopamine/Dopamine | 1.4 |
| Dimethylglycine | 1.5 |
| beta-D-Fructose 6-phosphate | 1.5 |
| L-Leucine | 1.5 |
| L-Tryptophan | 1.5 |
| L-Isoleucine | 1.5 |
| L-Histidine | 1.6 |
| beta-D-Fructose 1,6-bisphosphate | 1.6 |
| Adenine | 1.6 |
| Phosphoenolpyruvic acid | 1.6 |
| Succinic acid | 1.6 |
| alpha-D-Glucose 6-phosphate | 1.7 |
| D-Ribulose 5-phosphate | 1.7 |
| L-Homoserine | 1.7 |
| Acetyl-CoA | 1.7 |
| L-Phenylalanine | 1.8 |
| Oxidized glutathione | 1.8 |
| Histamine | 1.8 |
| Cytosine | 1.8 |
| Uridine triphosphate | 1.8 |
| L-Threonine | 1.9 |
| D-Glucose 1-phosphate | 1.9 |
| Sarcosine | 1.9 |
| Oxalacetic acid | 1.9 |
| Citric acid | 2.0 |
| Betaine | 2.0 |
| S-Adenosylhomocysteine | 2.0 |
| cis-Aconitic acid | 2.0 |
| Fumaric acid | 2.0 |
| Choline | 2.1 |
| Ornithine | 2.1 |
| L-Malic acid | 2.1 |
| L-Cysteine | 2.2 |
| Serotonin | 2.3 |
| L-Alanine | 2.4 |
| L-Asparagine | 2.7 |
| Glycine | 2.7 |
| L-Serine | 2.8 |
| L-Arginine | 2.8 |
| Betaine aldehyde | 2.9 |
| Glycolic acid | 3.0 |
| 2-Hydroxybutyric acid | 3.0 |
| Glutathione | 3.2 |
| L-Glutamic acid | 5.6 |
| Creatine | 7.9 |
